# Effect of the chemokine receptor CXCR7 on proliferation of carcinoma cells *in vitro* and *in vivo*

**DOI:** 10.1038/sj.bjc.6606002

**Published:** 2011-01-04

**Authors:** J Meijer, J Ogink, E Roos

**Affiliations:** 1 Division of Cell Biology, The Netherlands Cancer Institute, 1066CX Amsterdam, The Netherlands

**Retraction of**: *British Journal of Cancer* (2008) 99, 1493–1501; doi:10.1038/sj.bjc.6604727

After a thorough investigation, we have recently concluded that key results contributed by the first author cannot be reproduced. As the senior and corresponding author, I have therefore decided to retract this paper.

The co-author has been informed, and agrees with this decision. I emphasise that there is no doubt whatsoever about her contribution.

I sincerely apologise for the inconvenience this may have caused to readers of this journal.

Dr Ed Roos

Division of Cell Biology

The Netherlands Cancer Institute

